# Zinc-finger domains in metazoans: evolution gone wild

**DOI:** 10.1186/s13059-017-1307-y

**Published:** 2017-09-06

**Authors:** M. Mar Albà

**Affiliations:** 1 Evolutionary Genomics Group, Research Programme in Biomedical Informatics, Hospital del Mar Research Institute (IMIM)—Universitat Pompeu Fabra (UPF), Barcelona, Spain; 2 Catalan Institution for Research and Advanced Studies (ICREA), Passeig Lluís Companys, 08010 Barcelona, Spain

A new study uncovers a potential mechanism that may allow zinc-finger domains in metazoans to recognize and bind virtually any DNA sequence.

The human genome encodes about 700 proteins with Cys2-His2 zinc finger (C2H2-ZF) domains, the majority of which are likely to bind to DNA and regulate transcription. This makes C2H2-ZF the largest transcription factor (TF) family in our species [1], and the same is true in many other metazoans. By contrast, the genomes of more distant species, such as fungi and plants, encode a much more limited number of C2H2-ZFs.

A combination of evolutionary processes has resulted in a diverse range of C2H2-ZF proteins that show affinity for different DNA targets in metazoans [2]. In fact, human zinc-fingers may recognize a larger number of DNA sequences than all other human TFs combined [3]. This implies that zinc-fingers can potentially impact many diverse cellular processes. C2H2-ZF domains are short sequences that contain a beta-hairpin and an alpha-helix stabilized by a zinc ion, with only two cysteines and two histidines that are invariable. The rest of the amino acids can have almost any identity. Structural studies have shown that there are four positions in the alpha-helix that make direct contact with three or four bases in the DNA.

In a recent study published in *Genome Biology*, Najafabadi and colleagues [4] revealed why the C2H2-ZF protein domain is so frequent and diverse in metazoans but not in other eukaryotes, and how it is able to recognize so many different DNA targets, considering that only four residues directly contact the bases in DNA. By analyzing experimental data for thousands of zinc-finger domain sequences, they discovered that it is the contribution of residues that do not make direct contact with the bases that does the trick.

## Exuberant evolution of zinc-finger domains

Zinc-finger domains are rarely found alone; instead, they tend to form tandem arrays and sometimes they combine with different domains. This increases the length of the interaction surface and thus the number of different potential targets. The amount of redundancy in the binding sites is astonishing and it has been estimated that hundreds or even thousands of different C2H2-ZF sequences can recognize the same DNA triplet [5].

In general, transcription networks evolve through modifications of cis-regulatory sequences rather than through changes in the TFs. Mutations in the latter are often deleterious because they change the regulation of several genes at the same time. As a result, many developmental TFs are highly conserved across species. Zinc-finger containing proteins, however, do not appear to follow this general rule and have instead undergone very rapid diversification. At least some of these changes appear to have been adaptive [2], suggesting that this TF family has been an important driving force for evolutionary innovation.

Another interesting property of zinc-finger proteins is that, unlike other abundant TF families, they have undergone bursts of gene duplication at different evolutionary time points, such as at the base of the vertebrates or in the primate branch [6, 7]. Although the significance of this is not yet clear, it is tempting to speculate that it may have driven important species-specific adaptations.

## In search of a recognition code

A longstanding question is whether a zinc-finger–DNA recognition code exists. In other words, given the sequence of a zinc-finger domain, can we predict its DNA target? Deciphering such a code would be useful to identify the actual targets in the genome and to better understand the contribution of different amino acids to the binding mechanism.

To attempt to decipher the code, two studies [3, 5] used the one-hybrid system to estimate the DNA-binding affinities of thousands of natural zinc-finger domains. In this system, the interaction between the protein and the DNA sequence results in the expression of a reporter gene whose activity can be easily measured [8]. Persikov et al. [5] observed that not only could different zinc-finger sequences bind to the same DNA triplet, but the same zinc-finger could also recognize several different DNA targets. They also observed a negative relationship between the number of interactions and the strength of the binding, suggesting a trade-off between affinity and specificity. Najafabadi and colleagues [3] used information on the residues directly contacting the DNA bases to expand the zinc-finger–DNA recognition code. They observed that non-base-contacting amino acids also influenced the binding but the mechanism remained elusive. It was not until the study published in *Genome Biology* [4] that these data could be integrated into a more general model.

## The role of non-base-contacting residues in DNA binding

How does the relative abundance of the C2H2-ZF domain in metazoans compared to other eukaryotes affect the diversity of DNA targets? Najafabadi et al. [4] describe a novel approach to addressing this question by plotting the affinities of the domains present in 238 eukaryotes against all possible DNA triplets, using available one-hybrid data [5]. The species analyzed included both non-metazoans (mostly fungi and plants) and metazoans. By representing the data in this way, they could clearly see that, in contrast to metazoan zinc-fingers, non-metazoan zinc-fingers could only recognize a very limited subset of DNA motifs.

The researchers discovered that there were other fundamental differences between the two groups of organisms. The recognition code in non-metazoans was mostly determined by the base-contacting residues, with little influence from other amino acids in the sequence. However, in metazoans, both base-contacting and non-base-contacting residues were required for binding.

To unravel the complexity of zinc-fingers in metazoans, Najafabadi et al. [4] developed two separate models, one for each type of residue. The researchers used random forests, a machine-learning approach designed from a set of validated positive and negative cases. They observed that the non-base-contacting residues could discriminate between binding sites when the base-contacting residues were identical. By using molecular modeling of zinc-finger-DNA structures, they showed that these residues could contribute to the binding by forming hydrogen bonds with the DNA phosphate backbone. This is predicted to provide the necessary stability to the complex when the direct interactions with the bases are weak.

Najafabadi et al. [4] also analyzed the differences in DNA target binding between extant C2H2-ZFs and their inferred ancestral sequences. They concluded that the evolution of zinc-fingers was analogous to a kaleidoscope, with slight modifications of the amino acid sequence leading to dramatic changes in the preferred DNA targets, and with the combinations of motifs and targets appearing virtually unrestricted.

## Concluding remarks

The metazoan zinc-finger domain provides a fascinating example of exhaustive exploration of the sequence space, with only four residues being completely conserved and the rest being highly variable. As shown in the study by Najafabadi and co-workers [4], the binding to DNA depends both on residues that make direct contact with the bases in the DNA and on other residues that contact the phosphate backbone. As a consequence, the complete range of possible DNA triplets can potentially be recognized by C2H2-ZFs. In fact, the diversity of the contacts made by these domains is so high that several solutions exist in nature to bind to every DNA triplet.

The large-scale studies performed to date have focused on single domains; therefore, much remains to be learnt about the combinatorial effects of several C2H2-ZFs. New experiments will be required to validate the model and to understand how proteins with multiple domains behave. Another open question is why the fast evolution of zinc-fingers is not harmful, considering the potential of these domains to impact gene expression. Many C2H2-ZF proteins contain other types of domains, such as the Krüppel-associated box (KRAB), which represses the expression of endogenous retroviral elements. This may impose limits to the functionality of C2H2-ZFs, effectively taming their power.

## Abbreviations

C2H2-ZF: Cys2-His2 zinc finger
TF: Transcription factor
